# Morphological and molecular characterization of *Sarcocystis cooperii* n. sp. and related *Sarcocystis* species in three Colombian avian hosts

**DOI:** 10.14202/vetworld.2025.3255-3267

**Published:** 2025-10-31

**Authors:** Horwald Bedoya Llano, María Marín-Zapata, Cristina Úsuga-Monroy, Santiago Duque-Arias

**Affiliations:** Investigation Group (GINVER), School of Veterinary Medicine, Corporación Universitaria Remington, Medellín, Colombia

**Keywords:** avian parasitology, Colombia, *Coragyps atratus*, cytochrome c oxidase subunit 1, first internal transcribed spacer, phylogeny, *Pitangus sulphuratus*, *Sarcocystis cooperii* n. sp, *Vultur gryphus*

## Abstract

**Background and Aim:**

Apicomplexan parasites of the genus *Sarcocystis* exhibit complex two-host life cycles involving birds as both intermediate hosts (IH) and definitive hosts (DH). Despite Colombia’s exceptional avian diversity, knowledge of *Sarcocystis* species in its wild birds remains limited. This study aimed to characterize the morphology and genetic identity of *Sarcocystis* species infecting three Colombian birds, the Great Kiskadee (*Pitangus sulphuratus*), American black vulture (*Coragyps atratus*), and Andean condor (*Vultur gryphus*).

**Materials and Methods:**

Muscle samples from the three species were examined histologically using hematoxylin and eosin, periodic acid–Schiff, and toluidine blue staining. Transmission electron microscopy (TEM) was performed on *P. sulphuratus* samples to assess ultrastructural features. DNA was extracted and subjected to polymerase chain reaction amplification and sequencing of 18S ribosomal RNA, cytochrome c oxidase subunit 1, and internal transcribed spacer 1 region. Sequences were compared to GenBank data, and phylogenetic analysis was performed using the Maximum Likelihood method.

**Results:**

Two *Sarcocystis* species were identified. A novel species, *Sarcocystis cooperii* n. sp., was described from the Great Kiskadee, representing the first record of this species in South America. TEM revealed thin-walled (<1 μm), flat cysts with knob-like blebs corresponding to type 1a morphology. Molecular data showed 99.9% similarity with *Sarcocystis* sp. ex *Accipiter cooperii*, confirming its identity as *S. cooperii* n. sp., with the Cooper’s hawk acting as the putative DH. The *Sarcocystis* sp. detected in *C. atratus* and *V. gryphus* was genetically identical to isolates from Brazilian birds and closely related to *S. lari*, indicating a shared lineage among scavenging Cathartiformes.

**Conclusion:**

This study expands current knowledge of avian *Sarcocystis* diversity in the Neotropics, documenting *S. cooperii* n. sp. in *P. sulphuratus* and the first record of *Sarcocystis* sp. in two Cathartiformes species in Colombia. These findings underscore the ecological importance of synanthropic and scavenging birds in *Sarcocystis* transmission dynamics and highlight the need for further research on host–parasite relationships and potential conservation implications for endemic avifauna.

## INTRODUCTION

Members of the genus *Sarcocystis* are apicomplexan parasites characterized by a heteroxenous (two-host) life cycle and the formation of tissue cysts, known as sarcocysts, within the muscles of intermediate hosts (IHs). Definitive hosts (DHs) acquire infection by ingesting tissues that contain mature sarcocysts. Within the DH’s intestinal epithelium, the parasite undergoes sexual reproduction, forming oocysts or sporocysts that are subsequently shed with feces. IHs become infected after ingesting water or food contaminated with these sporocysts [1].

*Sarcocystis* infections are frequently reported in the skeletal muscles and intestinal tissues of birds throughout the Americas [2, 3]. Nevertheless, the taxonomic diversity of *Sarcocystis* species and the range of avian IHs involved in their life cycles remain poorly understood. Certain species, including *Sarcocystis calchasi*, *Sarcocystis falcatula*, and *Sarcocystis halieti*, display a broad host range and have been associated with severe or fatal infections in birds [4]. Conversely, *Sarcocystis* infections in raptors acting as DHs are typically subclinical. Despite Colombia’s status as one of the most avian-diverse countries globally, with approximately 1,941 bird species [5], studies addressing the occurrence and clinical significance of *Sarcocystis* spp. in its native birds are scarce.

Because many *Sarcocystis* species share overlapping morphological features, morphological examination alone is insufficient for accurate identification. Consequently, integrative approaches combining microscopy and molecular tools are essential. Morphological characterization of sarcocysts within IH tissues is complemented by molecular analysis targeting ribosomal RNA genes, the first internal transcribed spacer (ITS1), and the mitochondrial cytochrome c oxidase subunit 1 (cox1) loci. Among these, ITS1 has proven the most informative for distinguishing *Sarcocystis* species infecting avian IHs [1, 2, 4].

In Colombia, the Great Kiskadee (*Pitangus sulphuratus*), a widespread passerine of the family Tyrannidae distributed from the southern United States to central Argentina [6], was identified in this study as an IH for *Sarcocystis* sp. ex *Accipiter cooperii*. Two additional avian species, the American black vulture (*Coragyps atratus*) and the Andean condor (*Vultur gryphus*), both belonging to the order Cathartiformes, were also examined. The latter, listed as globally vulnerable by the IUCN, has experienced a marked population decline in recent decades [7]. The detection of *Sarcocystis* cysts within the muscles of these scavenging birds suggests that both species may serve as IHs in the parasite’s life cycle.

Despite the global distribution of *Sarcocystis* spp. and their recognized importance in avian parasitology, knowledge of their diversity, host range, and life cycle relationships in South American birds remains severely limited. Most existing studies on avian *Sarcocystis* have been conducted in Europe, North America, and parts of Asia, focusing mainly on domestic and synanthropic bird species or raptors as DHs. In contrast, the Neotropical region, particularly Colombia, one of the world’s most avian-diverse countries, has received little attention. Only a few molecular and histopathological surveys have been conducted, with most confined to isolated case reports or single-host observations.

Moreover, there is a lack of integrative studies that combine morphological, ultrastructural, and molecular data to confirm species identity and clarify evolutionary relationships among *Sarcocystis* spp. in wild birds. Because many species exhibit overlapping cyst morphology and variable host preferences, reliance solely on microscopy often leads to misidentification or underestimation of parasite diversity. Furthermore, the life cycles and host associations of *Sarcocystis* species involving wild passerines and scavenging birds (e.g., Cathartiformes) in South America remain largely uncharacterized. This represents a major gap in understanding the ecological and evolutionary dynamics of the genus in the Neotropics.

Another unexplored aspect is the potential role of synanthropic species, such as the Great Kiskadee (*P. sulphuratus*), in bridging parasite transmission between wild and urban environments. Similarly, scavenging species, such as the American black vulture (*C. atratus*) and the Andean condor (*V. gryphus*) may play an important but undocumented role in maintaining *Sarcocystis* cycles in natural ecosystems. Addressing these gaps is essential to clarify host–parasite relationships and to support wildlife disease surveillance and avian conservation efforts in the region.

This study aimed to perform comprehensive morphological and molecular characterization of *Sarcocystis* species infecting three avian hosts from Colombia, the Great Kiskadee (*P. sulphuratus*), American black vulture (*C. atratus*), and Andean condor (*V. gryphus*). Specifically, it sought to:

1. Identify and describe the structural and ultrastructural characteristics of *Sarcocystis* cysts within cardiac and skeletal muscles using light microscopy and transmission electron microscopy (TEM)
2. Determine the genetic identity and phylogenetic relationships of the detected *Sarcocystis* species by sequencing and analyzing three molecular markers (18S ribosomal RNA [18S rRNA], ITS1, and cox1)
3. Propose taxonomic classification of any novel or previously uncharacterized *Sarcocystis* species based on combined morphological and molecular evidence
4. Elucidate the potential host associations and epidemiological roles of the studied avian species within the life cycles of *Sarcocystis* spp., contributing to the broader understanding of parasite biodiversity and host ecology in Neotropical avifauna.

## MATERIALS AND METHODS

### Ethical approval

The collection and use of biological samples from wild birds were conducted in full compliance with Colombian and international ethical and biosafety standards. The protocol was reviewed and approved by the Bioethics Committee for Animal Research of the Facultad de Medicina Veterinaria, Corporación Universitaria Remington (CIBA-Acta No. 06–2021).

This study was authorized under the regulations of the National Environmental Licensing Authority (*Autoridad Nacional de Licencias Ambientales*) of Colombia, which mandates the registration of all wildlife specimens collected for research purposes. Necropsies were performed exclusively by certified veterinary pathologists and forensic veterinarians following the biosafety guidelines established by the World Organization for Animal Health (formerly OIE) [8] and the Veterinary Forensic Unit of Corporación Universitaria Remington. These procedures ensured adherence to national environmental laws and international standards on animal welfare, biosafety, and conservation ethics.

### Study period and location

From July 2021 to July 2024, three birds’ carcasses were collected opportunistically from three locations. The Medellín Botanical Garden, the Veterinary Forensic Unit of the Corporación Universitaria Remington, and the Regional Autonomous Corporation of Antioquia, all of them located in the department of Antioquia, Colombia.

### Study design and specimen collection

This investigation formed part of a broader research program funded by the Corporación Universitaria Remington, focusing on parasitic infections in wild avifauna. Three avian specimens were examined: The Great Kiskadee (*P. sulphuratus*), the American black vulture (*C. atratus*), and the Andean condor (*V. gryphus*).

- Great Kiskadee (*P. sulphuratus*): An adult male found dead in June 2023 at the Medellín Botanical Garden (6°16′15′′N, 75°33′51′′W); death was attributed to electrocution
- American black vulture (*C. atratus*): An adult male admitted to a wildlife rehabilitation center in April 2023 (6°8′3′′N, 75°16′28′′W) and deceased due to starvation following severe wing injury and flight incapacity
- Andean condor (*V. gryphus*): An adult female recovered in May 2021 from “Páramo del Almorzadero” Santander, Colombia (6°58′60′′N, 72°43′60′′W); necropsy indicated poisoning from organophosphates, carbamates, pyrethroids, and warfarin [7].

Specimens of *C. atratus* and *V. gryphus* were provided by regional environmental authorities specializing in wildlife management. Necropsy of *P. sulphuratus* was conducted on a fresh carcass, whereas the cathartid specimens were examined after thawing frozen carcasses. Muscle samples were collected from all individuals. For histological and morphological studies, one tissue fragment was fixed in 10% buffered formalin, while another was stored frozen without preservatives for molecular analysis.

### Histopathological and histochemical analysis

Ventricular (cardiac) muscle samples from all three species and pectoral muscle samples from *P. sulphuratus* and *C. atratus* were processed at the Animal Pathology Laboratory, Universidad de Antioquia. Tissues were dehydrated in graded ethanol, cleared in xylene, embedded in paraffin, and sectioned at 3 μm thickness. Sections were stained with hematoxylin–eosin to identify sarcocysts and assess tissue lesions, which were graded as absent, mild (<30%), moderate (30%–70%), or severe (>70%).

A histological reference collection of wild passerines and cathartids served as negative controls; all control tissues were confirmed negative for *Sarcocystis* spp. by polymerase chain reaction (PCR).

Histochemical stains, periodic acid–Schiff (PAS) and toluidine blue (TB), were used to visualize polysaccharides, glycoproteins, and acidic tissue components. PAS staining involved oxidation with periodic acid followed by Schiff’s reagent and hematoxylin counterstaining. TB staining was performed to enable metachromatic differentiation of sarcocyst structures. Details of histochemical protocols followed Marin-Zapata *et al*. [9]. Microscopic examination was performed using an Olympus CX23 microscope (Olympus, Tokyo, Japan), and photomicrographs were captured with a Leica EC3 digital camera (Leica Microsystems, Heerbrugg, Switzerland).

### TEM

Due to the limited availability of intact cysts, TEM analysis was performed exclusively on pectoral muscle tissue from *P. sulphuratus*. Tissue fragments (~2 mm^3^) from regions with the highest sarcocyst concentration were refixed in 2.5% phosphate-buffered glutaraldehyde for 24 h at 4°C and post-fixed in 1% osmium tetroxide for 1 h. Samples were dehydrated through a graded ethanol series, infiltrated with a 1:1 resin–acetone mixture, and embedded in SPURR resin (Electron Microscopy Sciences, Fort Washington, PA, USA).

Semithin (1 μm) sections were stained with TB to locate suitable regions, followed by ultrathin sectioning (80–100 nm) using a diamond knife on a Leica EM UC7 ultramicrotome. Sections were mounted on 300-mesh copper grids, stained with uranyl acetate and lead citrate, and examined under a JEOL 1400 Plus transmission electron microscope (Jeol, Tokyo, Japan).

### DNA extraction, PCR amplification, and sequencing

Genomic DNA was extracted from 25 mg to 50 mg of pectoral muscle tissue using the HigherPurity Tissue Genomic DNA Purification Kit (Canvax Biotech, Córdoba, Spain), following the manufacturer’s instructions, with final elution in 50 μL buffer. Molecular characterization targeted three loci: 18S rRNA, cox1, and the ITS1. PCR conditions and primer sets were used as described by Marin-Zapata *et al*. [9]. Each assay included a positive control (previously confirmed *Sarcocystis* DNA, GenBank OQ980375) and a negative control (molecular-grade water). All reactions were performed in duplicate using 50–100 ng of DNA template.

DNA purity and concentration were verified using a NanoDrop 2000 spectrophotometer (Thermo Fisher Scientific, USA). PCR products were purified and sequenced bidirectionally using the Sanger method (Macrogen, Korea).

### Molecular and phylogenetic analyses

Forward and reverse chromatograms were manually curated and aligned using ClustalW in BioEdit software package (version 7.7.1, https://bioedit. software.informer.com/7.7/) [10]. Consensus sequences were compared with homologous *Sarcocystis* spp. sequences available in GenBank using Basic Local Alignment Search Tool (http://blast.ncbi.nlm.nih.gov/Blast.cgi). Alignments were considered valid when query coverage was ≥99% for 18S rRNA and cox1, and ≥90% for ITS1.

Phylogenetic trees were constructed using the Maximum Likelihood method in MEGA-11 software (version 12.0.11, https://www.megasoftware.net/), applying the Hasegawa–Kishino–Yano substitution model [11, 12]. Tree robustness was tested using 1,000 bootstrap replicates. The final dataset comprised 122 sequences with 719 aligned nucleotide positions. Evolutionary analyses were performed in MEGA X software (version 10.2.2, https://www.megasoftware. net/) [13].

## RESULTS

### Overview of identified *Sarcocystis* species

Two *Sarcocystis* species were identified in this study: *Sarcocystis cooperii* n. sp. from the Great Kiskadee (*P. sulphuratus*) and *Sarcocystis* sp. from two Cathartiformes species, the American black vulture (*C. atratus*) and the Andean condor (*V. gryphus*).

The morphometric dimensions of the sarcocysts observed in each avian host are summarized in Table 1, while Table S1 (Supplementary data) presents the GenBank accession numbers and pairwise sequence comparisons of the newly obtained isolates.

**Table 1:** Morphometric characteristics of *Sarcocystis* cysts in three Colombian bird species.

Specimen	Length	Width	Wall thickness
*Pitangus sulphuratus*	244.66 µm	36.71 µm	0.34 µm
*Coragyps atratus*	51.5 µm	16.44 µm	0.82 µm
*Vultur gryphus*	45.68 µm	13.84 µm	0.45 µm

### Morphological and molecular characterization of *S. cooperii* n. sp. in the Great Kiskadee

#### Light microscopy (LM)

**Figure 1:**
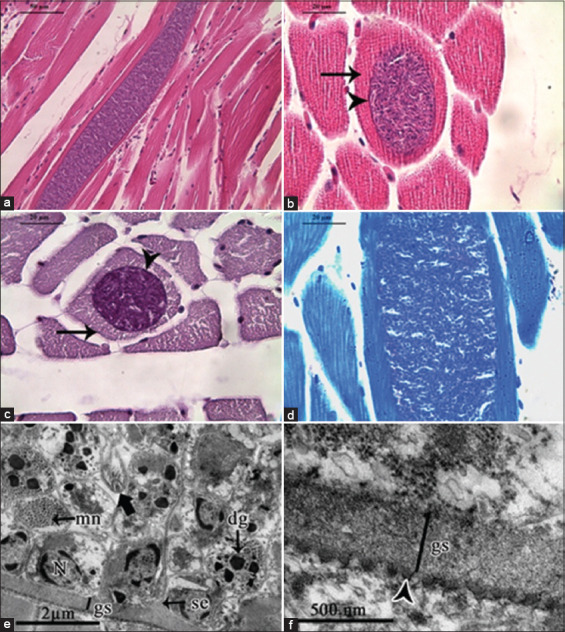
Microphotograph of *Sarcocystis* sp. in the skeletal muscle of *Pitangus sulphuratus*. (a) Longitudinal section of a sarcocyst with abundant bradyzoite and merozoites. Hematoxylin-eosin (HE). 400×. Bar = 50 μm. (b) Thin-walled sarcocyst (arrowhead) section within the host myocyte (arrow). HE. 1000×. Bar = 20 μm. (c) Thin-walled sarcocyst (arrowhead) in a myocyte with preserved basal lamina (arrow). Periodic acid–Schiff (PAS). 1000×. Bar = 20 μm. (d) Sarcocyst containing abundant zoites. Toluidine blue stain. 1000×. Bar = 20 μm. (e) Sarcocyst within a host cell. Granular substance (gs) with electrondense surface; septa (se) arising from granular substance; conoid (thick arrow) in a transverse-oblique section. Nucleus (N), dense granules (dg), and micronemes (mn) are shown. Bar = 2 μm. (f) Sarcocyst wall with knob-like blebs (arrowhead) and granular substance (gs). Bar = 500 nm.

LM revealed 227 sarcocysts within the cardiac and pectoral muscles of *P. sulphuratus*, covering an area of approximately 2.18 cm^2^ (Figures 1a-d). The cysts measured 86.96–607.68 μm in length (mean: 244.66 ± 182.9 μm) and 18.22–62.7 μm in width (mean: 36.71 ± 11.62 μm).

Histological sections revealed mild necrotic foci and limited hemorrhagic areas, accompanied by sparse lymphocytic infiltration, although these lesions were not associated with the presence of sarcocysts. The basal lamina of the host myocyte remained intact (Figure 1c), and the internal zoites exhibited uniform staining patterns (Figures 1b and d).

#### Ultrastructural features (TEM)

TEM (Figures 1e and f) demonstrated the presence of thin-walled sarcocysts containing metrocytes with distinct internal and external pellicle membranes. The wall consisted of a granular substance that formed septa, dividing the cyst interior into multiple compartments.

High-magnification views revealed a simple, flat cyst wall ornamented with knob-like blebs, each supported by a narrow stalk and bearing a rounded apical tip. The total wall thickness measured 0.34 μm, including villar protrusions (0.057 μm) and a granular layer (0.282 μm). These features correspond to wall type 1a, as described by Dubey *et al*. [1].

#### Molecular identification

Three genetic loci were successfully amplified and sequenced: 18S rRNA (809 bp; GenBank: PQ221914), cox1 (570 bp; PQ660003), and ITS1 (834 bp; PQ133336).

- The 18S rRNA gene exhibited 98.9% similarity to *S. halieti* (MF946587, LC830205) and to unnamed *Sarcocystis* sp. isolated from *A. cooperii* (KY348753), *Phalacrocorax carbo* (JQ733511), and *Columba livia* (GQ245670)
- The cox1 sequence showed 100% identity with *S. halieti*, *S. corvusi*, and *S. columbae*, and 99.82% similarity with *Sarcocystis* sp. ex *A. cooperii*, differing by a single-nucleotide polymorphism
- The ITS1 region shared 99.88% similarity with *Sarcocystis* sp. ex *A. cooperii* and 93%–96% similarity with *S. columbae*, *S. corvusi*, and *S. halieti* isolated from various avian hosts.

#### Taxonomic determination

Based on morphological features, ultrastructural analysis, and multi-locus phylogenetic congruence, the isolate from *P. sulphuratus* was established as a new species, *S. cooperii* n. sp., which shares its DH (*A. cooperii*) with the previously uncharacterized North American lineage.

#### Taxonomic summary

- Species: *S. cooperii* n. sp.
- IH: Great Kiskadee (*P. sulphuratus*)
- DH: Cooper’s hawk (*A. cooperii*)
- Type locality: Medellín, Colombia
- Etymology: Named after the Cooper’s hawk, the DH.
- Cyst dimensions: 87–607.7 × 18.2–62.7 μm (n = 20); thin-walled (<1 μm) with knob-like blebs (type 1a).
- GenBank accessions: PQ221914 (18S rRNA), PQ660003 (cox1), and PQ133336 (ITS1); reference sequences KY348753–KY348756 for comparison.
- Specimen deposition: Histological slides, TEM blocks, and extracted DNA archived under accession numbers M003, M036, and M038 at BioColección Uniremington, Medellín, Colombia.

### Morphological and molecular findings of *Sarcocystis* sp. in the American black vulture

#### Morphological features

**Figure 2:**
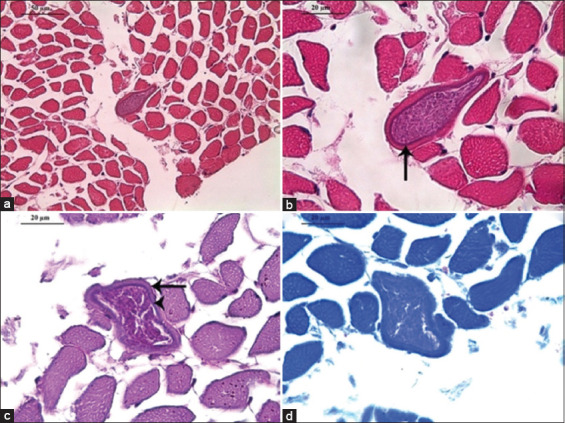
Microphotographs of *Sarcocystis* sp. in the skeletal muscle of *Coragyps atratus*. (a and b) Oblique sections of a thick-walled sarcocyst (black arrow) containing abundant zoites. Hematoxylin-eosin. 400×. Bar = 50 μm; and 1000×. Bar = 20 μm, respectively. (c) Thick-walled sarcocyst (arrowhead) in a myocyte with preserved basal lamina (arrow). Periodic acid–Schiff. 1000×. Bar = 20 μm. (d) Sarcocyst with abundant zoites. Toluidine blue stain. 1000×. Bar = 20 μm.

A single sarcocyst was observed in *C. atratus* muscle tissue sections (Figures 2a and b). The cyst measured 51.5 μm in length and 16.44 μm in width with a thin wall (0.82 μm). No significant inflammatory or degenerative changes were detected in adjacent tissues. The basal lamina was intact (Figure 2c), and the cyst contained numerous bradyzoites and metrocytes with pale staining (Figures 2b and d).

#### Molecular characterization

Amplified loci included 18S rRNA (810 bp; PQ221915), cox1 (570 bp; PQ660002), and ITS1 (810 bp; PQ133335).

- The 18S rRNA sequence showed 99.1%–99.3% similarity to *S. halieti* and several *Sarcocystis* spp. from avian hosts and marine mammals
- The cox1 gene exhibited complete identity with *S. lutrae*, *S. lari*, and *S. svanai*
- The ITS1 region shared 99.88% identity with *Sarcocystis* sp. from the kelp gull (*Larus dominicanus*) and 99.87% identity with *Sarcocystis* sp. previously isolated from *C. atratus* in Brazil, suggesting conspecificity.

### Morphological and molecular findings of *Sarcocystis* sp. in the Andean condor

#### Morphological features

**Figure 3:**
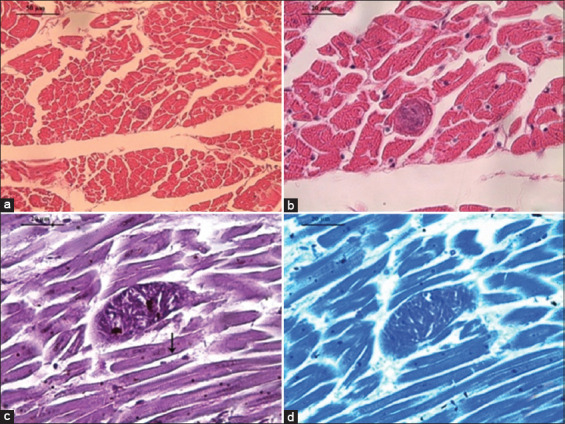
Microphotographs of *Sarcocystis* sp. in the cardiac skeletal muscle of *Vultur gryphus*. (a and b) Transverse sections of a sarcocyst containing abundant zoites. Hematoxylin-eosin. 400×. Bar = 50 μm; and 1000×. Bar = 20 μm, respectively. (c) Longitudinal section of a myocyte sarcocyst without a marked basal lamina. The basal lamina of another myocyte is shown for comparison (black arrow). Periodic acid–Schiff. 1000×. Bar = 20 μm. (d) Sarcocyst with abundant zoites, some of which stain more intensely. Toluidine blue stain. 1000×. Bar = 20 μm.

LM of *V. gryphus* muscle sections revealed a single thin-walled sarcocyst (Figures 3a and b) measuring 45.68 μm × 13.84 μm with a wall thickness of 0.45 μm. No apparent histopathological lesions were noted in the surrounding muscle tissue. The basal lamina was indistinct (Figure 3c), and numerous bradyzoites and metrocytes were present, some exhibiting more intense staining (Figure 3d).

#### Molecular characterization

Due to DNA degradation and limited sample volume, amplification of 18S rRNA and cox1 genes was unsuccessful. However, the ITS1 fragment (854 bp; PQ133334) was obtained and showed:

- 100% identity with the *C. atratus* isolate from this study
- 99.9% similarity with *Sarcocystis* sp. from *L. dominicanus*, and
- 89.3%–90.4% similarity with *S. lari*.

These findings indicate a close genetic relationship between *Sarcocystis* isolates from *C. atratus* and *V. gryphus*, and their South American seabird counterparts.

### Phylogenetic analysis

**Figure 4:**
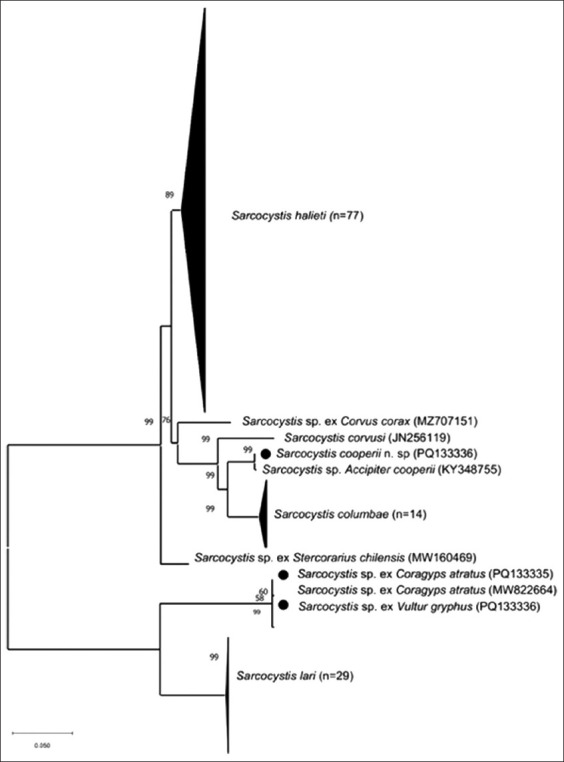
Phylogenetic tree of *Sarcocystis* spp. based on first internal transcribed spacer sequences. The number of branches represents bootstrap values >50% based on 1000 replicates. The black dot represents the sequences obtained in this study.

The ITS1-based phylogenetic tree (Figure 4) revealed distinct clustering patterns for the two *Sarcocystis* lineages.

- *S. cooperii* n. sp. grouped with *Sarcocystis* sp. ex *A. cooperii* with strong bootstrap support (99%), forming a sister clade to *S. columbae*
- *Sarcocystis* sp. isolates from *C. atratus* and *V. gryphus* clustered tightly with *Sarcocystis* sp. from *L. dominicanus* (Brazil), forming a separate lineage closely related to *S. lari*.

These results support the identification of *S. cooperii* n. sp. as a novel avian *Sarcocystis* species and indicate a shared evolutionary lineage among South American scavenging birds.

## DISCUSSION

### Overview and novel findings

**Figure 5:**
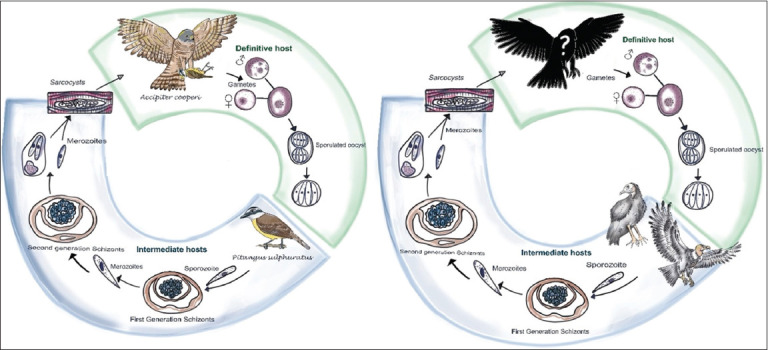
Schematic illustration of the proposed heteroxenous life cycle of *Sarcocystis cooperii* n. sp. (left) in the Great Kiskadee and *Sarcocystis* sp. (right) in the American black vulture and Andean condor. *S. cooperii* n. sp. transmission occurs when Cooper’s hawk consumes infected Passeriformes (*Pitangus sulphuratus*). The transmission of *Sarcocystis* sp. presumably occurs when prey birds act as definitive hosts and consume infected Cathartiformes or other bird species.

Herein, we explain the role of the Great Kiskadee (*P. sulphuratus*) as an IH in the life cycle of *Sarcocystis*, where the Cooper’s hawk (*A. cooperii*) serves as the DH, thus contributing to the understanding of the genus epidemiology involving avian hosts. We also report, for the 1^st^ time, the occurrence of *Sarcocystis* sp. in two Cathartidae species from Colombia (*C. atratus* and *V. gryphus*) (Figure 5). In the only previous study from Colombia, *S. falcatula* was reported in a clinically healthy toucan [9].

### Morphological observations and diagnostic value of light microscopy

LM revealed thin-walled *Sarcocystis* cysts, a finding consistent with previous studies by Marin-Zapata *et al*. [9], Prakas *et al*. [14], Sneideris *et al*. [15]. However, LM alone was insufficient to determine the species or genus in the vulture samples analyzed. Despite these limitations, LM remains an important preliminary diagnostic approach that complements molecular techniques by providing morphological context for cyst identification.

### Ultrastructural characterization and taxonomic significance

TEM of *P. sulphuratus* sarcocysts revealed crucial ultrastructural characteristics, including short digitiform protrusions extending from a thin granular layer. These microstructures are essential for species-level identification within the genus *Sarcocystis*, as they are not discernible by conventional LM. The observed features correspond with those reported by Lindsay *et al*. [16], who described the sexual stages of the parasite but did not characterize cyst morphology in *A. cooperii*. This finding highlights the indispensable role of TEM in differentiating *Sarcocystis* species with similar wall morphologies but distinct ultrastructural patterns [2, 9].

### Molecular markers and phylogenetic delineation

Several studies have shown that 18S rRNA and cox1 markers are not sufficiently variable to allow reliable differentiation among avian *Sarcocystis* species [2, 17, 18]. In contrast, the ITS1 locus has proven highly informative for species discrimination in bird–bird life cycles. Based on ITS1 phylogenetic analysis, *S. cooperii* n. sp. forms a distinct clade related to avian *Sarcocystis* species with similar host associations (Figure 4).

These include *S. columbae*, which uses *Accipiter* hawks as DHs and European pigeons (*Columba palumbus*), herring gulls (*Larus argentatus*), common gulls (*Larus canus*), and black-headed gulls (Larus ridibundus) as IHs [14, 19–22]; *Sarcocystis* sp. ex *Corvus corax*, *Sarcocystis* sp. isolate Skua-2016-CH, and *S. corvusi*, which infect the common raven (*C. corax*), skuas (*Stercorarius chilensis*), and jackdaws (*Corvus monedula*), respectively [17, 23, 24]. The DHs for the latter two species remain unknown. Recently, Juozaitytė-Ngugu *et al*. [25] confirmed the role of the common buzzard (*Buteo buteo*), Eurasian goshawk (*Accipiter gentilis*), and Eurasian sparrowhawk (*Accipiter* nisus) as DHs for *Sarcocystis* sp. ex *C. corax*. Finally, *S. halieti* has been shown to possess a broad range of avian IHs, with the white-tailed sea eagle (*Haliaeetus albicilla*) serving as its DH [4].

### Cathartiformes isolates and feeding ecology

Morphological and molecular findings for *Sarcocystis* sp. from *V. gryphus* and *C. atratus* correspond to isolates reported from Brazilian birds [2, 26]. The DH for this lineage remains unknown. This group comprises scavenging birds that likely acquire infection through ingestion of infected muscle tissues from other avian species harboring the parasite’s asexual stages.

Phylogenetic analysis based on ITS1 sequences revealed a close relationship between this *Sarcocystis* species and *S. lari* (Figure 4). Although *S. lari* has been associated with *H. albicilla* and several Corvidae species in Europe as DHs [25, 27], it clusters phylogenetically with species that use mammals as DHs rather than birds of prey [4]. Considering that *S. lari* has only been detected in Europe, while the Cathartiformes-associated *Sarcocystis* occurs in South America, we hypothesize that this divergence reflects geographical isolation. Geographic variation among *Sarcocystis* populations has been documented previously by Sazmand *et al*. [18], Prakas *et al*. [28], Prakas *et al*. [29]. Further studies are required to clarify the phylogenetic and ecological basis of this separation.

### DH associations and host range flexibility

Phylogenetic analysis of the ITS1 region revealed two major host-associated groups:

1. Group I – species for which birds of prey act as DHs, including *S. cooperii* n. sp; and
2. Group II – species from Cathartiformes, potentially involving mammalian DHs that remain unidentified.

The capacity of *Sarcocystis* species to exploit diverse DH taxa is well documented. For instance, *S. rileyi* forms macrocysts in ducks, using members of the Mephitidae, Mustelidae, Canidae, and Procyonidae families as DHs [30]. Thus, the Cathartiformes isolates may also involve alternative mammalian DHs.

### Ecological and conservation implications

The detection of *Sarcocystis* in the Andean condor (*V. gryphus*), an endangered species, without evident macroscopic or microscopic lesions suggests potential tolerance or coexistence that merits further study in a conservation context. Both *V. gryphus* and *C. atratus* occupy crucial ecological roles as scavengers, although their feeding strategies differ. While condors primarily consume large carcasses, black vultures exhibit a more generalist and opportunistic diet, including smaller carcasses, fruits, insects, reptiles, and human-associated waste [31–33]. These behaviors may facilitate their exposure to *Sarcocystis* and enhance their importance in maintaining parasite transmission cycles in wild and anthropogenic environments.

### Transmission dynamics of *S. cooperii* n. sp.

The Great Kiskadee (*P. sulphuratus*), a synanthropic passerine with a varied diet of arthropods, small vertebrates, mollusks, and fruits, likely acquires *S. cooperii* n. sp. by ingesting water or food contaminated with sporocysts released by *A. cooperii*. As an abundant and ecologically adaptable species, *P. sulphuratus* serves as a potential bridge host between wildlife and urban habitats [34, 35]. Frequent interactions with domestic and peridomestic animals could enable the cross-ecosystem flow of *Sarcocystis*. To date, however, no *Sarcocystis* species transmitted by birds have been implicated in human infection.

### Research priorities and future directions

This study underscores the need for expanded molecular and ecological investigations of *Sarcocystis* spp. in Neotropical birds. Future research should aim to:

- Identify the DHs of Cathartiformes-associated *Sarcocystis* spp.
- Clarify phylogeographic relationships among *Sarcocystis* populations; and
- Assess potential ecological and conservation implications in endangered avifauna.

Integrated morphological, ultrastructural, and molecular approaches across multiple trophic levels will be crucial for elucidating the complex life cycles of *Sarcocystis* in Neotropical ecosystems.

## CONCLUSION

This study provides the first morphological and molecular evidence of *S. cooperii* n. sp. infecting the Great Kiskadee (*P. sulphuratus*) in Colombia, with the Cooper’s hawk (*A. cooperii*) identified as the likely DH. The species was characterized by thin-walled cysts (<1 μm) with knob-like blebs of wall type 1a, and ITS1 phylogenetic analysis placed it within a distinct clade of *Sarcocystis* species that exhibit bird–bird life cycles. In addition, *Sarcocystis* sp. was identified in two Cathartidae species, the American black vulture (*C. atratus*) and the Andean condor (*V. gryphus*), representing the first such records from Colombia. Molecular comparisons revealed a close genetic relationship (99%–100%) with *Sarcocystis* sp. previously reported from scavenging birds in Brazil and a phylogenetic association with *S. lari*. No macroscopic or histopathological lesions were observed in the infected condor and vulture tissues, suggesting a subclinical or tolerant host–parasite interaction.

These findings expand the current understanding of avian *Sarcocystis* diversity in the Neotropics and highlight the ecological and epidemiological importance of synanthropic and scavenging birds in maintaining parasite transmission cycles. The Great Kiskadee, an abundant and ecologically flexible species, may act as an epidemiological bridge between wild and urban ecosystems through exposure to sporocyst-contaminated water or prey, while scavengers such as vultures and condors could serve as reservoir or accidental IHs due to their carrion-based feeding behavior. The identification of *S. cooperii* n. sp. contributes valuable reference sequences for future molecular diagnostics and supports the establishment of wildlife parasitic surveillance frameworks in Latin America.

The main strength of this study lies in the integration of histological, ultrastructural, and multi-locus molecular analyses, allowing a taxonomically robust species characterization. It also documents the first evidence of *Sarcocystis* infection in Cathartidae in Colombia and provides insights into parasitic tolerance in an endangered species, the Andean condor. However, the limited number of specimens, opportunistic nature of sampling, and partial genetic data from degraded tissues constrained life-cycle confirmation and broader epidemiological inference.

This work broadens the biogeographical and host-range knowledge of *Sarcocystis* spp. in South America, revealing new ecological connections among passerines, raptors, and scavengers. The description of *S. cooperii* n. sp. underscores the hidden diversity of avian *Sarcocystis* and the need for expanded, integrated studies combining morphological, molecular, and ecological approaches to elucidate transmission dynamics and assess conservation and One Health implications within Neotropical ecosystems.

## AUTHORS’ CONTRIBUTIONS

HBL: Conceptualization, study design, writing – review and editing, writing – original draft, supervision, and formal analysis. MMZ: Methodology and sample collection. CUM: Formal analysis and methodology. SDA: Formal analysis, methodology, project administration, and writing – review and editing. All authors have read and approved the final version of the manuscript.
